# MDM2 inhibitor induces apoptosis in colon cancer cells through activation of the CHOP-DR5 pathway, independent of p53 phenotype

**DOI:** 10.3389/fphar.2025.1508421

**Published:** 2025-04-08

**Authors:** Manman Lu, Yingli Ren, Sijia Feng, Shenggen Wang, Weiyue Xia, Baoru Gu, Yuhou Shen, Aimin Yue, Na Li, Yongxi Zhang, Jiateng Zhong

**Affiliations:** ^1^ Department of Oncology, The Third Affiliated Hospital of Xinxiang Medical University, Xinxiang, China; ^2^ Department of Pathology, The First Affiliated Hospital of Xinxiang Medical University, Xinxiang, China; ^3^ College of Medicine, Henan Polytechnic University, Jiaozuo, China; ^4^ Henan Province Engineering Technology Research Center of Tumor Diagnostic Biomarkers and RNA Interference Drugs, The Third Affiliated Hospital of Xinxiang Medical University, Xinxiang, China; ^5^ Department of Abdominal Surgical Oncology Ward 2, Xinxiang Central Hospital, Xinxiang, China; ^6^ Department of Abdominal Surgical Oncology Ward 2, The Fourth Clinical College of Xinxiang Medical University, Xinxiang, China

**Keywords:** nutlin-3a, apoptosis, extrinsic apoptotic pathway, ER stress, colorectal cancer

## Abstract

**Introduction:**

Murine double minute 2 (MDM2), a key negative regulator of p53, forms a feedback loop with p53 to drive tumor progression, including colorectal cancer. Nutlin-3a, an MDM2 inhibitor, induces apoptosis in wild-type p53 tumors, but its effects on p53-mutated cancers and potential p53-independent apoptotic mechanisms remain unclear.

**Methods:**

We investigated Nutlin-3a's effects on colon cancer cells with varying p53 phenotypes. Endoplasmic reticulum (ER) stress-associated CHOP was detected and knocked down to explore mechanisms. In vitro and in vivo experiments assessed Nutlin-3a's synergy with 5-fluorouracil and TRAIL.

**Results:**

Nutlin-3a activated caspase-8-dependent extrinsic apoptosis in colon cancer cells via DR5 upregulation, independent of p53 status. ER stress and CHOP activation mediated DR5 induction, driven by calcium release. Combined Nutlin-3a treatment enhanced sensitivity to 5-fluorouracil and TRAIL *in vitro* and *in vivo* through caspase-8 pathway activation.

**Discussion:**

These findings reveal a novel p53-independent apoptotic mechanism of Nutlin-3a involving ER stress and death receptor signaling. This pathway highlights Nutlin-3a's potential as an adjuvant therapy for colon cancer, even in p53-mutated tumors, by enhancing chemotherapeutic efficacy through extrinsic apoptosis.

## 1 Introduction

Colorectal cancer (CRC) is the third most widespread malignant tumor in the world, second only to lung cancer and breast cancer (Cao C. et al., 2020). According to the cancer statistics for 2022 from the International Agency for Research on Cancer (IARC), colorectal cancer is the second most diagnosed cancer type globally, accounting for approximately 9.6% of all cancer cases and causing approximately 9.3% of cancer deaths (Bray et al., 2024). At present, the treatment of colorectal cancer is still based on surgery combined with radiotherapy and chemotherapy, and the sensitivity of chemotherapy is one of the main factors affecting the survival of patients (Vaidya et al., 2020). Studies show that patients with chemotherapy tolerance often have high recurrence and metastasis rates, which is the main cause of death in colorectal cancer patients, especially in the late stage (Vodenkova et al., 2020). The commonly used chemotherapeutic drugs for colorectal cancer include 5-fluorouracil (5-FU) and oxaliplatin, but the specificity of these drugs is limited, and they are ineffective in advanced colorectal cancer patients (Tan et al., 2019). Although some classic targeted drugs such as regorafenib are used in the treatment of colorectal cancer, with the prolongation of drug use cycle, there are obvious drug resistance phenomena (Arai et al., 2019). It is urgent to find new targeted and combined drug strategies to prolong the life of patients with colorectal cancer.

MDM2 (murine double minute 2) is a common E3 ubiquitin ligase; like other E3 ligases, MDM2 participates in the regulation of various cellular biological behaviors by targeting ubiquitinated protein substrates (Wallace et al., 2006; Humpton et al., 2021; Ying et al., 2016). Among these substrates, the direct relationship between MDM2 and tumor suppressor gene p53 has gained extensive attention (Shangary and Wang, 2008; Lessel et al., 2017). On the one hand, MDM2 can induce its ubiquitination and degradation by targeting p53 protein; on the other hand, this binding reduces the binding ability of p53 to other downstream molecules and induces p53 nuclear export. These factors lead to the decline of p53 level and its ability to regulate target genes, and finally, p53 loses its inhibitory effect on tumor (Wang et al., 2017). Based on these studies, MDM2 has been identified as a recognized oncogene, which is involved in the regulation of the occurrence and development of a variety of cancers (Mastronikolis et al., 2020; Sookaromdee and Wiwanitkit, 2019). Therefore, research workers have carried out extensive research and development on MDM2 inhibitors, among which nutlin-3a is the first effective and selective small molecule MDM2 antagonist (Vassilev et al., 2004; da Mota et al., 2016; Lauria et al., 2010). *In vivo* administration of nutlin-3a can effectively inhibit the tumor growth of SCID mice, which, for the first time, proves that it is feasible to activate wild-type p53 through a pharmacological inhibitor of p53/MDM2 interaction *in vivo* (Vassilev et al., 2004). At present, whether nutlin-3a has a killing effect on p53-mutant cancer cells is not clear, and whether there is a mechanism independent of p53 phenotype has not been reported.

The main mechanism of anticancer therapies is to kill cancer cells by inducing apoptosis (Tan et al., 2019; Hanahan and Weinberg, 2000). Apoptosis is mainly regulated by two signaling pathways, the intrinsic pathway (mitochondrial pathway and endoplasmic reticulum stress-induced apoptotic pathway) and the extrinsic pathway (death receptor) (Derakhshan et al., 2017; Sano and Reed, 2013). The mitochondrial apoptotic signaling pathway is mainly regulated by the Bcl-2 protein family (Edlich, 2018). Proapoptotic protein member (such as Bax) activates apoptosis by reducing mitochondrial membrane potential and releasing cytochrome C, which is located between the inner and outer membranes of mitochondria (Birkinshaw and Czabotar, 2017). Endoplasmic reticulum (ER) stress plays a dual role in cell death. When the degree of ER stress is mild, it protects cells from death. However, when ER stress transitions, it can induce stress-related apoptosis by activating CHOP (C/EBP homologous protein) (Chevet et al., 2015). The activation of the extrinsic pathway is mainly through the binding of proapoptotic ligands and TNF family receptors. It has been reported that the binding of TRAIL (tumor necrosis factor-related apoptosis-inducing ligand) and DR5 (death receptor 5) activates caspase-8 and induces death receptor-related apoptosis (Deng and Shah, 2020; Twomey et al., 2015). Some reports show that DR5 can also be activated by p53 under DNA damage and by CHOP in response to ER stress (He et al., 2016; Hu et al., 2019).

At present, MDM2 inhibitor nutlin-3a is mainly used in the treatment of pediatric tumors and hematologic malignancies. Few studies have been reported on the inhibitory effect of nutlin-3a on colorectal cells, and the mechanism of nutlin-3a acting on the death receptor signaling pathway is still unclear. In this study, we show that nutlin-3a can induce apoptosis in colorectal cancer cells, not through the mitochondrial apoptotic pathway but through the activation of DR5 by ER stress apoptotic protein CHOP, which then activates the extrinsic apoptotic pathway. In addition, the administration of nutlin-3a increased the sensitivity of colon cancer cells to clinical chemotherapeutic drugs 5-FU and TRAIL. In this study, we provide a basis for the clinical use of nutlin-3a in the treatment of colorectal cancer and also provide a new idea for the combination with traditional chemotherapy drugs to overcome drug resistance.

## 2 Materials and methods

### 2.1 Cell culture and treatment

The human colorectal cancer cell lines, including RKO, HCT116, CT26 (WT p53), and SW480 and CACO2 (mutant p53), were maintained in RPMI-1640 (Invitrogen, United States) supplemented with 10% FBS (Gibco) and 1% penicillin–streptomycin (Invitrogen, United States). All cells were cultured in a 37°C incubator at 5% CO_2_. For drug treatment, the selection of nutlin-3a doses was guided by preliminary experiments that determined the IC50 values for various cell lines and the findings from the study by Crane et al. (2015). In this study, we showed that nutlin-3a effectively inhibited cell proliferation in 15 different cell lines at varying concentrations, including 1 μM, 5 μM, 10 μM, 25 μM, 50 μM, and 70 μM. Based on these observations, we selected intermediate concentrations (35 µM, 50 µM, and 75 µM) that had a significant effect on both types of cells as the concentrations used for our experiments to examine their effects on apoptosis and ER stress-related molecules. For the long-term drug treatment colony formation assay, HCT116 and RKO cells were treated with varying concentrations of nutlin-3a (2 μM or 4 μM) or 0.1% DMSO as a control for a duration of 8 days. Cells were seeded overnight at 60%–70% confluence and were either treated or not with the indicated concentration of drugs or combination of drugs. Nutlin-3a (Sigma), TRAIL (MCE), BAPTA (MCE), and 5-fluorouracil (MCE) were diluted with DMSO (Sigma).

### 2.2 CCK-8 assay

The indicated cells were seeded in 96-well plates at a density of 1.2 × 10^4^ cells/well. After 24 h of incubation, the cells were treated with the indicated concentration of nutlin-3a alone or in combination with TRAIL and 5-fluorouracil in 100 μL complete medium for another 20 h. Cell viability was evaluated using the Cell Counting Kit-8 (CCK-8) assay (Dojindo, Japan) according to the manufacturer’s instructions. Each assay was conducted in triplicate and repeated three times.

### 2.3 Quantitative real-time PCR

Total RNA was isolated from cells using the TRIzol reagent (Invitrogen, United States), and 1 µg of total RNA was reverse transcribed using a RevertAid First Strand cDNA Synthesis Kit (Thermo Fisher) in a final volume of 20 µL. The resulting cDNA (1 µL) was diluted 1:10 and was used as a template for real-time PCR amplification using the UltraSYBR Mixture (High ROX) (cwbiotech) in a final reaction volume of 10 µL. The real-time PCR was performed in triplicate for each sample, and GAPDH was used as a reference for normalization. Results were represented by the threshold cycle value (Ct), and the 2^−ΔΔCt^ method was used to analyze the relative changes in gene expression. The primers used for real-time PCR are listed in Supplementary Table S1.

### 2.4 Apoptosis analysis

Apoptosis was analyzed by flow cytometry according to the manufacturer’s instruction. For flow cytometry analysis, cells were plated in a 6-well plate to incubate for 24 h and treated with the indicated concentration drugs alone or in combination with TRAIL/5-FU for an additional 20 h at 37°C. The cells were then collected in 100 μL Annexin V-FITC binding buffer and stained with Annexin V and propidium iodide (Dojindo, Japan). The percentage of apoptosis was analyzed by FlowJo software.

### 2.5 Western blotting assay

Total protein was first extracted from drug-treated cells with cell lysis buffer. Cell lysis buffer (20–30 µg) was then separated by 10% SDS-PAGE electrophoresis under 120 V, transferred onto the polyvinylidene difluoride (PVDF) membrane, and then blocked in 5% skimmed milk for 1 h. Then, the PVDF membrane was incubated with primary antibodies diluted with 5% skimmed milk overnight at 4°C and followed with secondary antibodies for 1 h. The immunoblot bands of aim protein were detected with horseradish peroxidase (HRP), and ImageJ software was used for quantitative analysis. The following antibodies were used for protein detection: tubulin (cat#2146s), cleaved caspase-3 (cat#9661s), Bax (cat#5023s), cytochrome C (cat#4280s), Bcl-2 (cat#3498s), DR5 (cat#8074s), cleaved caspase-8 (cat#9496s), XBP1s (cat#12782s), Bip (cat#3183s), Phosph-eIF2a (cat#3597s), ATF-4 (cat#11815s), CHOP (cat#2895s), and MDM2 (cat#86934).

### 2.6 Clonogenic survival assay

In the exponential phase of growth, cells were plated in 6-well plates at approximately 300 cells/well in complete media and were treated with the indicated concentration of nutlin-3a by the third day. After 24 h, the medium was replaced with complete media and then cultured in a 37°C incubator at 5% CO_2_ for 8 days. The colonies that formed were stained by crystal violet staining, followed by being fixed in 4% paraformaldehyde solution.

### 2.7 Transfection and viral transduction

For transfection, 5 × 10^5^ RKO cells were seeded in 60-mm plates overnight and were transfected with Lipofectamine 2000 (Invitrogen, United States) in Opti-MEM medium (Invitrogen, United States) according to the manufacturer’s instructions. Transfection was carried out 24 h prior to drug treatment using 600 pmol of small-interfering RNA (siRNA). Small-interfering RNA duplexes were synthesized from GenePharma (Shanghai) and include the following: CHOP (5′-GCACAGCUAGCUGAAGAGAdTdT-3′), DR5 (5′-AAGACCCUUGUGCUCGUUGUCdTdT-3′), and control scramble siRNA. Sh-caspase-8 and sh-MDM2 cell lines of HCT116 were constructed via virus system (Lenti-Easy Packaging System, Genechem, Shanghai). For viral transduction, 7 × 10^5^ 293 T cells were plated in 60-mm plates overnight and co-transfected with lentiviral vector, along with Lenti-Easy Packaging Mix for 8 h by using Lipofectamine 2000. The viral supernatant from the transfected cells was collected at 48 h and 72 h, respectively, after transfection and then filtered with 0.22-μm PES membrane filters (Millipore). Media with progeny after filtration were freshly used to infect HCT116 cells in the presence of Enhanced Infection Solution (Genechem, Shanghai). After infection, the cells were treated with 2 μg/mL puromycin (Sigma-Aldrich) for 5–8 days to eliminate the uninfected cells. Western blot was used to verify the effection of transfection as well as transduction.

### 2.8 Ca^2+^ fluorescence imaging

Fluo-4 AM, a calcium fluorescence probe, was used to quantify cellular Ca^2+^ concentrations. A total of 5 × 10^5^ cells were inoculated in a 24-well plate and treated the next day with the indicated concentration of nutlin-3a for 4 h, 8 h, and 12 h, respectively. After washing three times with phosphate-buffered saline (PBS) (HyClone), the treated cells were incubated with Fluo-4 AM for 30 min at 37°C in the dark, and then the green fluorescence was photographed using fluorescent microscopy and analyzed by flow cytometry.

### 2.9 Xenograft studies

In this study, we utilized 25 5-week-old female nude mice (BALB/c-nu) obtained from Charles River Laboratory Center. The mice were housed in individually ventilated cages (IVCs) in a controlled sterile environment with a temperature range of 22°C–24°C and relative humidity of 40%–60%. The light cycle was maintained on a 12-hour light/dark schedule.

The primary aim of the study was to investigate xenograft tumors derived from HCT116 cells. To induce tumor formation, HCT116 cells (5 × 10^6^) were resuspended in 200 μL of serum-free medium and injected subcutaneously into the left inguinal region of each mouse using a 26-gauge needle. Following the implantation, the tumors were allowed a 7-day acclimatization period for growth.

After 7 days, tumor-bearing mice were randomly assigned to five treatment groups (n = 5 per group) using a random number generator. For our *in vivo* experiments, we adopted an oral dosing regimen of nutlin-3a at 150 mg/kg administered twice daily, based on the study by Tovar et al. (2006). This study demonstrated that oral administration of nutlin-3a at doses ranging from 100 to 200 mg/kg effectively inhibited tumor growth in xenograft models. The selected dose was chosen to ensure therapeutic relevance while minimizing potential adverse effects, providing an appropriate balance for evaluating the efficacy and safety of nutlin-3a in our mouse models. The treatment regimen lasted for 15 days, during which the following interventions were applied: intraperitoneal injection of PBS (100 μL/day), intraperitoneal injection of 5-FU (23 mg/kg/day), intratumoral injection of TRAIL (1 µg every 2 days), or a combination of these treatments with nutlin-3a administered orally (150 mg/kg twice daily) for the duration of the study. The mice were randomly assigned to five treatment groups: group 1: intraperitoneal injection of PBS (100 μL/day), group 2: intraperitoneal injection of 5-FU (23 mg/kg/day), group 3: intratumoral injection of TRAIL (1 µg every 2 days), group 4: combination of 5FU (23 mg/kg/day) and nutlin-3a administered orally (150 mg/kg twice daily), and group 5: combination of TRAIL (1 µg every 2 days) and nutlin-3a administered orally (150 mg/kg twice daily).

The changes in health status were monitored daily, and the body weight of the mice was recorded every 3 days. The longest (denoted as “a”) and perpendicular (denoted as “b”) dimensions of the tumors were measured using calipers every 3 days. Tumor volumes (v) for each mouse were then calculated using the following formula: v = 0.5×a × b2. Three hours after the final treatment, the mice were anesthetized using isoflurane to ensure humane and painless procedures. Following anesthesia, blood samples were collected *via* orbital bleeding, and then the mice were euthanized by cervical dislocation. Subsequently, tumors were harvested for further analysis.

### 2.10 Hematoxylin and eosin (HE) staining for tissue samples

Tissue collection and preparation: following euthanasia, liver and kidney samples were carefully harvested from the mice to avoid damage to the tissue architecture. The tissues were gently rinsed twice with phosphate-buffered saline (PBS) to remove residual blood and contaminants. Samples were then fixed in 10% neutral buffered formalin for 24 h at room temperature to preserve the tissue structure.

Tissue processing: fixed tissue samples were dehydrated sequentially through graded alcohol solutions: 70% ethanol for 1 h, 80% ethanol for 1 h, 95% ethanol for 1 h, and, finally, 100% ethanol for 1 h (repeated twice). Dehydrated tissues were cleared in xylene (three changes, 10 min each) to prepare them for embedding. The cleared tissues were embedded in molten paraffin wax and allowed to solidify, ensuring proper orientation for sectioning.

Sectioning and slide preparation: paraffin blocks were trimmed, and 5-μm-thick tissue sections were cut using a rotary microtome. Sections were carefully placed onto pre-cleaned, positively charged glass slides to improve adhesion. Slides were dried overnight at 37°C to ensure proper adherence of the tissue sections to the slides.

Deparaffinization and rehydration: slides were heated at 65°C for 1 h to melt the paraffin and improve tissue adherence. Deparaffinization was performed by immersing the slides in xylene for 10 min (repeated twice). Sections were rehydrated sequentially through a descending series of ethanol concentrations: 100% ethanol for 5 min (two changes), 95% ethanol for 5 min, and 70% ethanol for 5 min, and finally, they were rinsed in distilled water for 5 min.

Hematoxylin staining: tissue sections were stained with hematoxylin for 5–10 min to highlight nuclei. Excess hematoxylin was removed by rinsing the slides under running tap water for 5 min. Sections were then dipped in 0.3% acid alcohol (1% hydrochloric acid in 70% ethanol) for a few seconds to differentiate the staining. Slides were rinsed again under running tap water for 5 min and dipped in ammonia water (0.2% ammonium hydroxide solution) to stain the nuclei blue, followed by rinsing again in water.

Eosin staining: counterstaining was performed by immersing the slides in eosin solution for 1–2 min to stain cytoplasm and extracellular matrix components. Excess eosin was removed by rinsing in distilled water.

Dehydration and mounting: stained sections were dehydrated through an ascending ethanol series: 70% ethanol for 1 min, 95% ethanol for 1 min, and 100% ethanol for 2 min (repeated twice). Dehydrated sections were cleared in xylene (two changes, 5 min each). Coverslips were mounted onto the slides using neutral resin, ensuring bubble-free application.

Microscopic observation: prepared slides were allowed to dry at room temperature overnight. Sections were examined under a light microscope to assess tissue morphology and identify any pathological changes.

### 2.11 Mouse serum alanine aminotransferase (ALT) testing

Blood collection: at the conclusion of the treatment regimen, mice were anesthetized to minimize stress and discomfort during blood collection. Blood was collected via orbital bleeding using a capillary tube. Care was taken to avoid contamination and ensure sufficient sample volume (∼200–300 μL per mouse). Collected blood samples were transferred into clean, labeled microcentrifuge tubes (preferably serum-separating tubes) and allowed to coagulate at room temperature for 30 min to facilitate clot formation.

Serum separation: coagulated blood samples were centrifuged at 2,000 rpm (approximately 500×g) for 10 min at 4°C. The supernatant (serum) was carefully transferred into new, pre-labeled microcentrifuge tubes without disturbing the blood clot. The serum samples were immediately stored at −80°C for future analysis or processed directly to measure ALT levels.

Preparation of reagents: the Alanine Aminotransferase (ALT) Assay Kit (C009-1-1, Nanjing Jiancheng Bioengineering Institute, Nanjing) was used according to the manufacturer’s instructions. Reagents were equilibrated to room temperature before use. The standard solution was diluted to prepare a series of known concentrations for the ALT standard curve.

ALT assay procedure: serum samples and ALT standards were loaded into a 96-well microplate. Typically, 50 μL of serum or standard solution was added per well. In total, 50 μL of the substrate solution (provided in the kit) was added to each well and mixed thoroughly by gentle pipetting. The reaction mixture was incubated at 37°C for the time specified in the kit instructions (commonly 15–30 min). Following incubation, 50 μL of the color development reagent was added to each well to terminate the reaction and develop color.

Optical density measurement: the microplate was read using a microplate reader at an absorbance wavelength of 505 nm. Each sample was measured in triplicate to ensure accuracy and reproducibility. Blank wells containing only reagents without serum were included as controls.

Calculation of ALT activity: the optical density (OD) values of the standards were plotted to generate a standard curve. The OD values of the serum samples were compared to the standard curve to determine the corresponding ALT (GPT) activity, which was expressed in U/L.

### 2.12 TUNEL assay for apoptosis

Subcutaneous tumor tissues in nude mice after drug action were removed and fixed in 4% tissue fixative for 24 h and then cut and placed straight in a tissue embedding box and labeled accordingly. The tumor tissues were then dehydrated, embedded, and sectioned, and the apoptosis of the tumor tissues was detected using the TUNEL apoptosis assay kit; DAPI was used to stain the nucleus.

### 2.13 Detection of caspase-8 enzyme activity

The cells were collected and washed twice with PBS. Lysis buffer was added, and the cells were lysed on ice for 15 min. After centrifugation at 16,000 g for 15 min, 50 μL of the supernatant was taken. Then, 10 μL of Ac-IETD-pNA (2 mM) and 40 μL of the detection buffer were added. It was mixed well and incubated at 37°C for 80 min. The absorbance was measured at A405. The absorbance of the sample at A405, after subtracting the absorbance of the blank control, represents the absorbance generated by pNA produced from the catalytic activity of caspase-8 in the sample.

### 2.14 Statistics analysis

Statistical analyses were carried out using SPSS 20.0 and GraphPad Prism Ⅷ software. We used the unpaired two-tailed Student’s t-test to calculate p-values and determine the statistical significance of the differences between the samples. The results are presented as the mean + or ±standard deviation (+SD or ±SD). We express the p-value as *, **, ***, or n.s. to indicate statistical significance, with the respective cutoff values of *p* < 0.05, *p* < 0.01, *p* < 0.001, or no significance. When making four-group comparisons, we analyzed the data using 1-factor- or 2-factor-ANOVA. If the ANOVA was overall significant, we used a *post hoc* Student’s t-test to compare pairs of samples.

## 3 Results

### 3.1 MDM2 inhibitor nutlin-3a induces apoptosis of colon cancer cells through activating the death receptor pathway and not the mitochondrial apoptotic pathway

**FIGURE 1:**
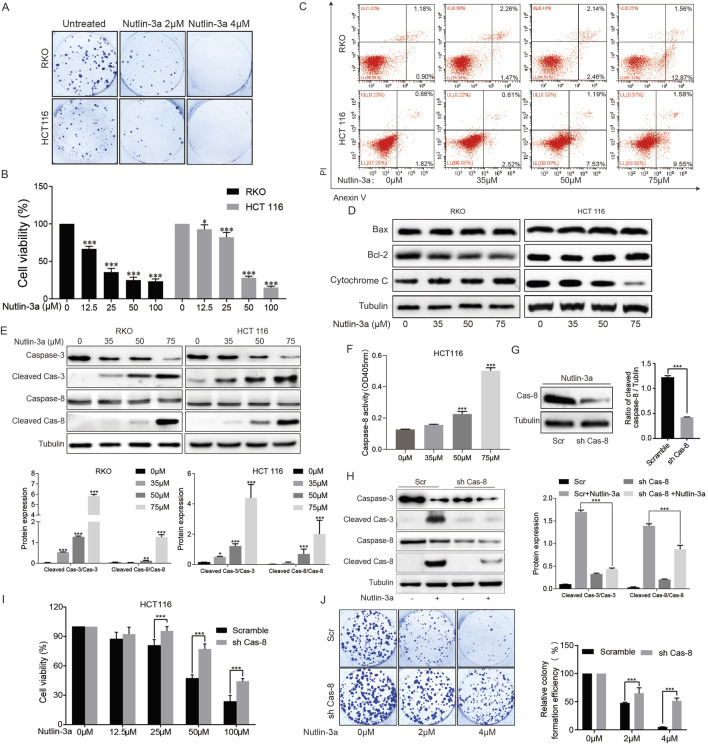
MDM2 inhibitor nutlin-3a inhibits survival and proliferation and activates apoptosis in CRC cells, and caspase-8 was required for nutlin-3a-induced apoptosis. **(A)** Colony formation assay of HCT116 and RKO cells treated with different concentrations of nutlin-3a (2 or 4 μM) or 0.1% DMSO for 8 days, and the attached cells were stained with 1% crystalline violet and photographed. The clones were counted. Data are presented as the mean ± SD. **(B)** HCT116 and RKO cells were treated with different concentrations of nutlin-3a (12.5, 25, 50, or 100 μM) or 0.1% DMSO for 20 h, and the cell viability was determined by CCK-8 assays. Data are presented as the mean ± SD. **(C)** Apoptosis rate of HCT116 and RKO cells was assessed after treatment with nutlin-3a (35, 50, or 75 μM) or 0.1% DMSO for 20 h by flow cytometry. **(D)** HCT116 and RKO cells were treated with different concentrations of nutlin-3a (35, 50, or 75 μM) or 0.1% DMSO for 20 h, and Bax, Bcl-2, and cytochrome C proteins were analyzed by Western blot. **(E)** After being treated with different concentrations of nutlin-3a (35, 50, or 75 μM) or 0.1% DMSO for 20 h, cleaved caspase-3 and cleaved caspase-8 proteins in HCT116 and RKO cells were analyzed by Western blot. Data are presented as the mean ± SD. **(F)** After being treated with different concentrations of nutlin-3a (35, 50, or 75 μM) or 0.1% DMSO for 20 h, a caspase-8 assay kit was used to measure the activity of caspase-8. **(G)** Caspase-8 knockdown confirmed by Western blotting in HCT116 cells stably expressed scramble shRNA or caspase-8 shRNA. Data are presented as the mean ± SD. **(H)** Caspase-8 knockdown and control HCT116 cells were treated with nutlin-3a (55 μmol/L) for 20 h, and cleaved caspase-3 and cleaved caspase-8 proteins were detected by Western blotting. Data are presented as the mean ± SD. **(I)** Caspase-8 knockdown and control HCT116 cells by shRNA were treated with nutlin-3a (12.5, 25, 50, and 100 μM) or 0.1% DMSO for 20 h, and the cell viability was determined by CCK-8 assays. **(J)** Colony formation assay of caspase-8 knockdown and control HCT116 cells by shRNA after being treated with different concentrations of nutlin-3a (2 or 4 μM) or 0.1% DMSO for 8 days, and the attached cells were stained with 1% crystalline violet and photographed. The clones were counted. Data are presented as the mean ± SD.

To explore the effects of nutlin-3a in colon cancer cells, cell proliferation and viability were detected in HCT116 and RKO cells. The results showed that nutlin-3a can significantly inhibit the proliferation of colon cancer cells and induce a decrease in the cell survival rate (Figures 1A, B). Building on the study by Crane et al. (2015), we conducted dose–response experiments and observed varying degrees of inhibition in cell proliferation at different nutlin-3a concentrations. The results of flow cytometry analysis showed that different concentrations of nutlin-3a can significantly induce apoptosis in both types of cells (Figure 1C).

To clarify the mechanism of nutlin-3a-induced apoptosis of colon cancer cells, we first examined the classical mitochondrial apoptosis signaling pathway. We used Western blotting to detect the key molecules of the mitochondrial apoptosis signaling pathway, Bcl-2 family members, Bax, and cytochrome C. The results showed that administering different concentrations of nutlin-3a caused no changes in Bcl-2, Bax, and cytochrome C (Figure 1D), suggesting that the endogenous mitochondrial apoptosis signaling pathway may not be directly involved in the occurrence of nutlin-3a-induced cell apoptosis. Next, we turned our attention to another key signaling pathway for apoptosis regulation, the death receptor signaling pathway. We detected death receptor-related caspase-8, and Western blotting results showed that nutlin-3a can significantly induce the expression of cleaved caspase-8, especially at high concentrations (Figure 1E). Additionally, we measured the activity of caspase-8. The results indicate that as the concentration of nutlin-3a increases, the activity of caspase-8 significantly increases (Figure 1F). To clarify the role of the death receptor signaling pathway in nutlin-3a-induced cell apoptosis, we constructed shRNA caspase-8 and found that inhibiting caspase-8 in cells can significantly reduce the activation of the apoptotic effector molecule caspase-3 and the inhibition of cell proliferation caused by nutlin-3a (Figures 1G–J).

The above results suggest that nutlin-3a can induce cell apoptosis through the death receptor signaling pathway, but it is not yet clear how the death receptor signaling pathway plays a regulatory role.

### 3.2 Nutlin-3a activates death receptor signaling by upregulating DR5

**FIGURE 2:**
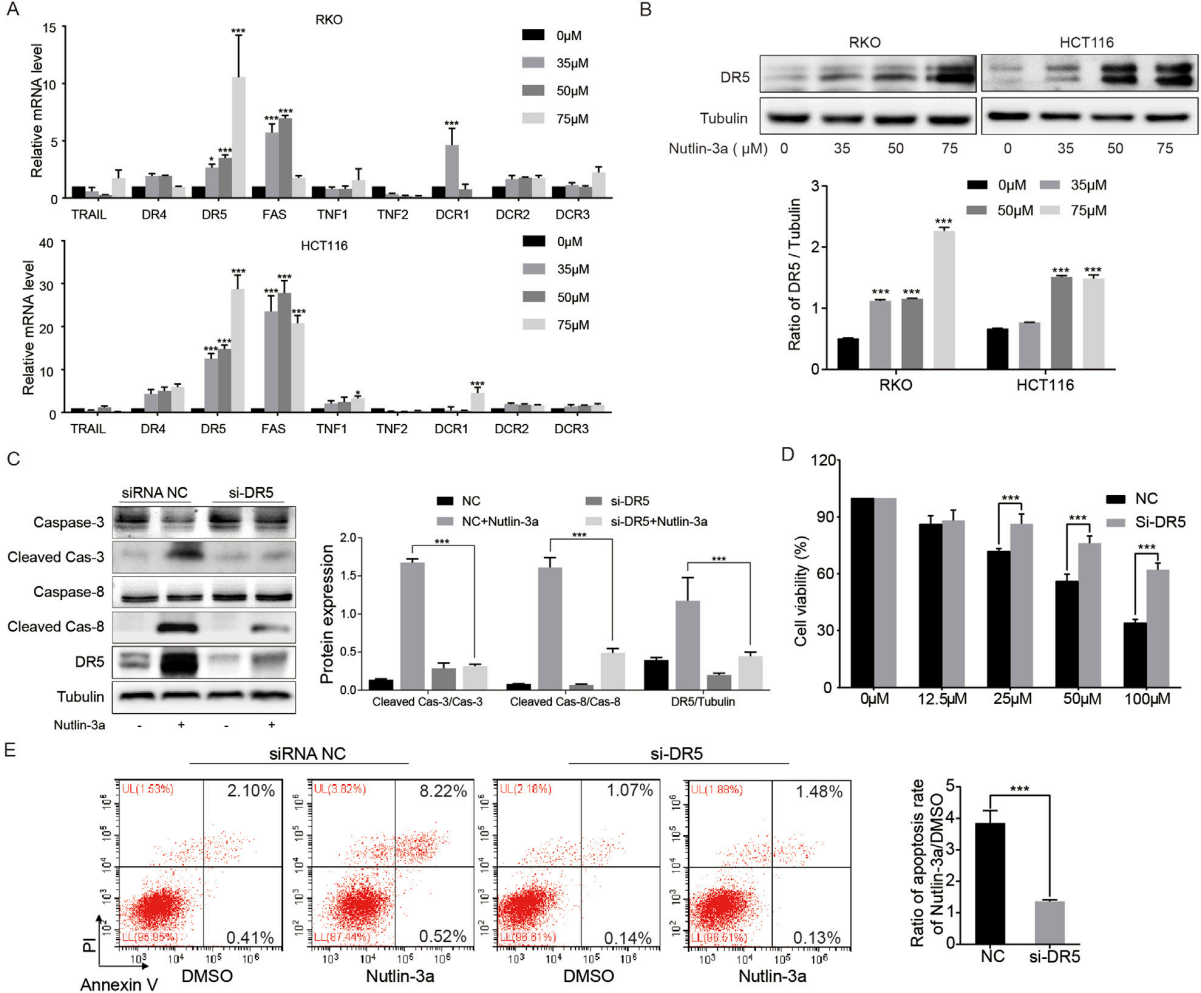
DR5 was required for nutlin-3a-induced apoptosis. **(A)** HCT116 and RKO cells were treated with different concentrations of nutlin-3a (35, 50, or 75 μM) or 0.1% DMSO for 20 h, and mRNA levels of TRAIL, DR4, DR5, FAS, TNF1, TNF2, DCR1, DCR2, and DCR3 were analyzed by real-time PCR. **(B)** DR5 protein was analyzed by Western blotting after being treated with nutlin-3a (35, 50, and 75 μM) or 0.1% DMSO for 20 h in HCT116 and RKO cells. **(C)** DR5 knockdown and control HCT116 cells by siRNA were treated with nutlin-3a (55 μmol/L) for 20 h, and cleaved caspase-3, cleaved caspase-8, and DR5 proteins were analyzed by Western blotting. Data are presented as the mean ± SD. **(D)** DR5 knockdown and control HCT116 cells by siRNA were treated with nutlin-3a (55 μmol/L) for 20 h, and cell viability was detected by the CCK-8 assay. **(E)** DR5 knockdown and control HCT116 cells by siRNA were treated with nutlin-3a (55 μmol/L) for 20 h, and the apoptosis rate was analyzed by flow cytometry. Data are presented as the mean ± SD. *, *p* < 0.05; **, *p* < 0.01; ***, *p* < 0.01.

In order to clarify the regulation of nutlin-3a on the death receptor pathway, qRT-PCR detected a panel of extrinsic apoptosis regulators in colon cancer cells. The results indicated that DR5 was the common receptor with significantly increased expression in HCT116 and RKO cells (Figure 2A). DR5 protein detection showed that the results were consistent with mRNA (Figure 2B). To determine whether nutlin-3a-induced activation of the death receptor pathway is dependent on DR5, we used siRNA to knock down the expression of DR5 in HCT116 cells and found that siRNA DR5 significantly inhibited nutlin-3a-induced activation of caspase-8 and caspase-3 (Figure 2C). In addition, inhibition of DR5 expression can significantly alleviate the nutlin-3a-induced decrease in cell survival and increase in apoptosis (Figures 2D, E). To sum up, nutlin-3a-induced death receptor-dependent apoptosis occurs through DR5.

### 3.3 Nutlin-3a upregulation of DR5 and cleaved caspase-8 independent of the p53 phenotype

**FIGURE 3:**
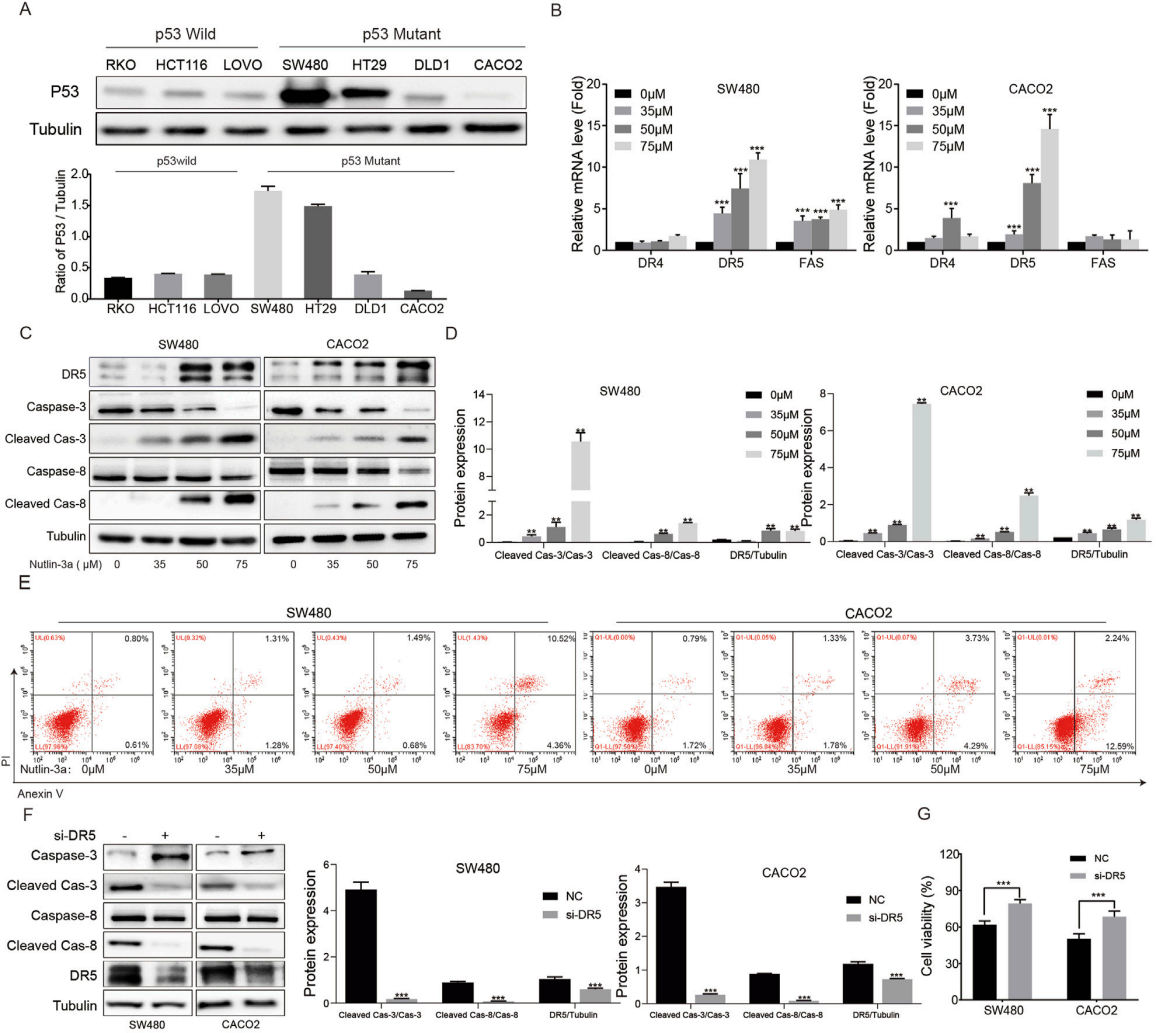
Nutlin-3a-induced apoptosis of p53-mutant colon cancer cells via DR5 and is independent of the p53 phenotype. **(A)** p53 protein was analyzed by Western blotting in p53 wild-type colon cancer cell lines (RKO, HCT116, and LOVO) and p53 mutant colon cancer cell lines (SW480 and CACO2). **(B)** p53 mutant colon cancer cell lines (SW480 and CACO2) were treated with different concentrations of nutlin-3a (35, 50, or 75 μM) or 0.1% DMSO for 20 h, and the changes in the mRNA levels of DR4, DR5, and FAS were analyzed by real-time PCR. *, *p* < 0.05; **, *p* < 0.01; ***, *p* < 0.01. **(C)** p53 mutant colon cancer cell lines (SW480 and CACO2) were treated with nutlin-3a (35, 50, and 75 μM) or 0.1% DMSO for 20 h, and the changes of DR5, cleaved caspase-3, and cleaved caspase-8 proteins were detected by Western blot. **(D)** Ratio of cleaved caspase-3 vs. caspase-3 and cleaved caspase-8 vs. caspase-8 in SW480 and CACO2 was calculated. Data are presented as mean + SD. **P < 0.01; n.s., not significant, by 1-factor ANOVA with a *post hoc* t-test. **(E)** Apoptosis rate of SW480 and CACO2 cells was assessed after treatment with nutlin-3a (35, 50, or 75 μM) or 0.1% DMSO for 20 h by flow cytometry. **(F)** DR5 knockdown and control HCT116 cells by siRNA were treated with 55 μmol/L nutlin-3a for 20 h, and DR5, cleaved caspase-3, and cleaved caspase-8 were analyzed by Western blotting. **(G)** DR5 knockdown and control cells (SW480 and CACO2) were treated with nutlin-3a (55 μmol/L) for 20 h, and cell viability was detected by the CCK-8 assay. *, *p* < 0.05; **, *p* < 0.01; ***, *p* < 0.01.

Inhibition of MDM2 induces p53 activation, and MDM2 inhibitor-induced apoptosis is often dependent on wild-type p53. To determine whether the activation of the nutlin-3a-induced upregulation of DR5 and cleaved caspase-8 is dependent on the p53 phenotype, colon cancer cells with wild-type and mutant p53 and different basal expression levels of p53 were used for further experiments (Figure 3A). qRT-PCR results showed that nutlin-3a could also significantly increase the level of DR5 mRNA in SW480 and CACO2 cells (Figure 3B). Protein detection results showed that nutlin-3a could increase the expression of DR5, cleaved caspase-8, and cleaved caspase-3 in p53 mutant SW480 and CACO2 cells (Figure 3D). To evaluate whether different p53 phenotypes affect nutlin-3a-induced apoptosis, we performed grayscale analysis of cleaved caspase-3 and cleaved caspase-8 levels in p53 wild-type cells (RKO and HCT116) (Figure 1E) and p53 mutant cells (SW480 and CACO2) (Figure 3C) after treatment with different concentrations of nutlin-3a over the same period. The analysis results from Supplementary Figures S1A, S1B indicate that following treatment with 35 μM and 50 μM nutlin-3a, there were no significant differences in the levels of cleaved caspase-3 and cleaved caspase-8 between p53 wild-type cells (RKO and HCT116) and p53 mutant cells (SW480 and CACO2). At a concentration of 75 μM, a notable difference was observed. RKO cells exhibited higher cleaved caspase-3 levels than CACO2, whereas HCT116 showed higher levels than SW480. In contrast, SW480 had elevated cleaved caspase-3 levels. Treatment with 50 μM and 75 μM nutlin-3a resulted in significant changes in cleaved caspase-8, but this difference was not influenced by whether the cells were p53 wild-type or mutated. Meanwhile, we compared the apoptosis levels detected by flow cytometry in p53 wild-type cells (RKO and HCT116) (Figure 1C) and p53 mutant cells (SW480 and CACO2) (Figure 3E) after treatment with different concentrations of nutlin-3a. The results (Supplementary Figure S1C) show that nutlin-3a-induced apoptosis did not differ between p53 wild-type and p53 mutant cells. These may be caused by varying degrees of tolerance of cells to high concentrations of drugs.

In addition, compared with the control group, DR5 siRNA could significantly reduce the activation of cleaved caspase-8 and cleaved caspase-3 induced by nutlin-3a (Figure 3F). Simultaneous inhibition of DR5 in SW480 and CACO2 cells alleviated the nutlin-3a-induced decrease in cell viability (Figure 3G). These data demonstrate that the nutlin-3a activated death receptor apoptotic pathway of colon cancer cells was independent of the p53 wild-type or mutant cells.

### 3.4 Nutlin-3a induces DR5 expression through endoplasmic reticulum stress-mediated CHOP activation

**FIGURE 4:**
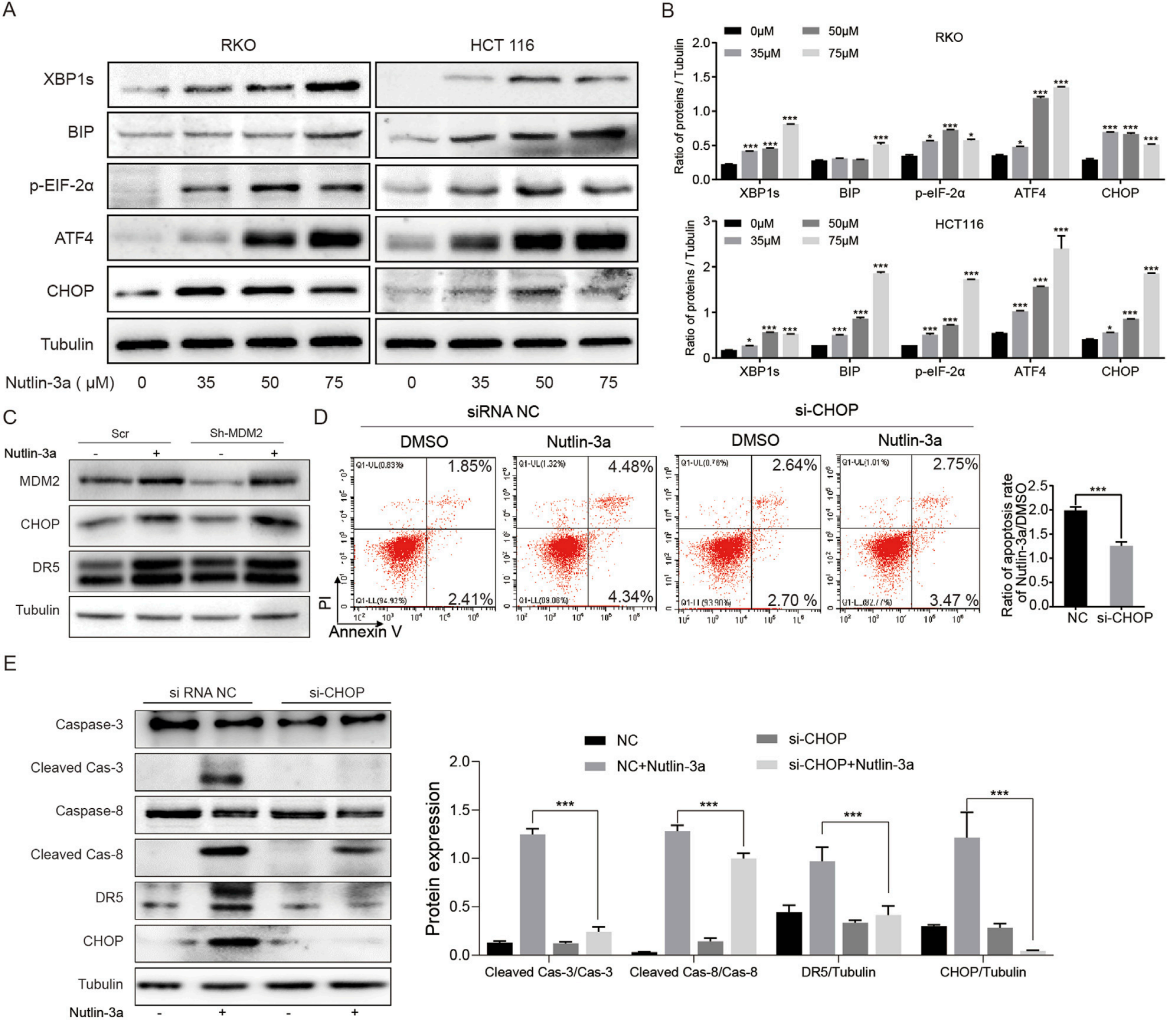
Nutlin-3a induces DR5 expression and apoptosis in colon cancer cells through endoplasmic reticulum stress-mediated CHOP activation. **(A, B)** HCT116 and RKO cells were treated with different concentrations of nutlin-3a (35, 50, or 75 μM) or 0.1% DMSO for 20 h, and XBPIs, BIP, ATF4, p-EIF-2α, and CHOP proteins were analyzed by Western blot. Data are presented as the mean ± SD. **(C)** MDM2 knockdown and control HCT116 cells by shRNA were treated with 55 μmol/L nutlin-3a for 20 h, and MDM2, CHOP, and DR5 were analyzed by Western blotting. **(D)** CHOP knockdown and control HCT116 cells by siRNA were treated with nutlin-3a (55 μmol/L) for 20 h, and the apoptosis rate was analyzed by flow cytometry. The percentages of apoptotic cells are presented in the histogram as the mean values ± SD. *, *p* < 0.05; **, *p* < 0.01; ***, *p* < 0.01. **(E)** CHOP knockdown and control HCT116 cells by siRNA were treated with nutlin-3a (55 μmol/L) for 20 h, and cleaved caspase-3, cleaved caspase-8, DR5, and CHOP proteins were analyzed by Western blotting. Data are presented as the mean ± SD.

The activation of DR5 is caused by the activation of p53 under DNA damage or CHOP under ER stress (Deng and Shah, 2020). The above results have excluded the influence of the p53 phenotype, and the next experiment focuses on CHOP regulation. Nutlin-3a can significantly activate the ER stress signaling pathway and then upregulate CHOP expression (Figures 4A, B). To investigate whether nutlin-3a-induced ER stress is related to MDM2, we constructed sh-RNA to knock down MDM2 in HCT116 cells and treated them with nutlin-3a. We found that ER stress could still be induced, suggesting that it is seemingly independent of its direct target MDM2 (Figure 4C). The results of flow cytometry showed that inhibiting the expression of CHOP could significantly reduce the increase of apoptosis rate induced by nutlin-3a (Figure 4D). Further experiments showed that knockdown of CHOP in HCT116 cells could significantly inhibit the upregulation of DR5, caspase-8, and caspase-3 induced by nutlin-3a (Figure 4E). These results show that nutlin-3a induced upregulation of DR5 through ER stress-induced activation of CHOP.

### 3.5 Nutlin-3a mediated ER stress by inducing the release of calcium ions in ER

**FIGURE 5:**
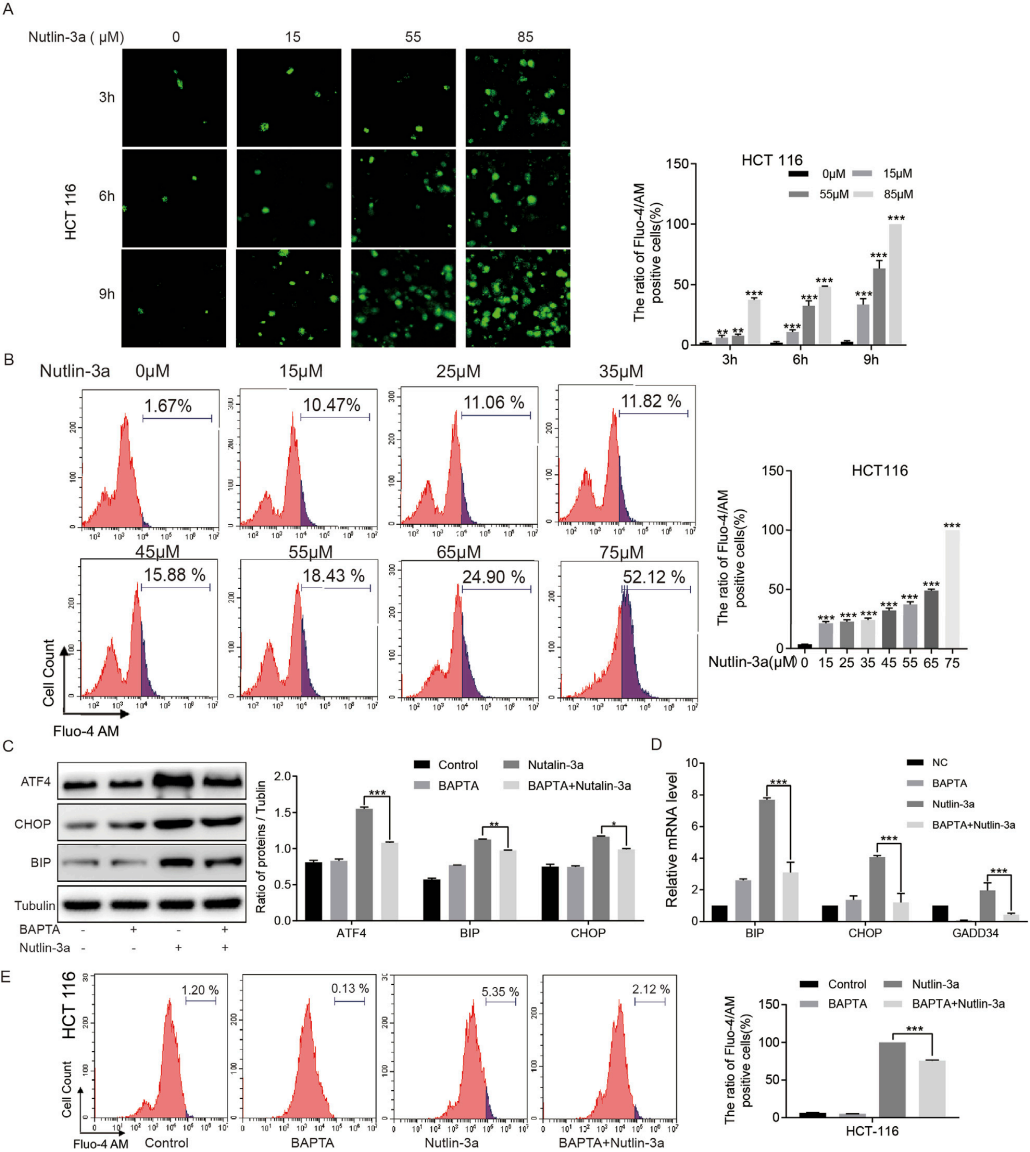
Nutlin-3a mediated ER stress by inducing the release of calcium ions in ER. **(A)** HCT116 cells were treated with different concentrations of nutlin-3a (15, 55, or 85 μM) or 0.1% DMSO for 3 h, 6 h, and 9 h, and intracellular Ca^2+^ was tagged with Fluo-4 AM and photographed using fluorescent microscopy. The ratio of Fluo-4 AM-positive cells are presented in the histogram as the mean ± SD. **(B)** HCT116 cells were treated with different concentrations of nutlin-3a (15, 25, 35, 45, 55, 65, 75, or 75 μM) or 0.1% DMSO for 12 h, and intracellular Ca^2+^ was marked with Fluo-4 AM. The ratio of Fluo-4 AM-positive cells was analyzed by flow cytometry. The percentages of apoptotic cells are presented in the histogram as the mean ± SD. **(C)** HCT116 cells were treated with BAPTA-AM (10 μM), nutlin-3a (45 μM), or their combination for 20 h, and ATF4, CHOP, and BIP proteins were analyzed by Western blot. Data are presented as the mean ± SD. **(D)** HCT116 cells were treated with BAPTA-AM (10 μM), nutlin-3a (45 μM), or their combination for 20 h, and the changes in the mRNA levels of BIP, CHOP, and GADD34 were analyzed by real-time PCR. **(E)** HCT116 cells were treated with BAPTA-AM (10 μM), nutlin-3a (45 μM), or their combination for 20 h, and intracellular Ca^2+^ was marked with Fluo-4 AM. The ratio of Fluo-4 AM-positive cells was analyzed by flow cytometry. The percentages of apoptotic cells are presented in the histogram as the mean ± SD. *, *p* < 0.05; **, *p* < 0.01; ***, *p* < 0.01.

In order to clarify the reason of the occurrence of ER stress in colon cancer cells caused by nutlin-3a, we detected the concentration of cytoplasmic calcium ion, which is closely related to ER stress. The results of fluorescent microscopy showed that nutlin-3a significantly increased the concentration of calcium ions in the cytoplasm of colon cancer cells (Figure 5A). Meanwhile, flow cytometry assays showed consistent results (Figure 5B). The effect of calcium ions on nutlin-3a-induced ER stress was further clarified using calcium chelating agents (BAPTA). The result showed that BAPTA can reduce the expression of induced ER stress-related proteins and genes induced by nutlin-3a (Figures 5C–E).

### 3.6 Death receptor-mediated chemosensitization by nutlin-3a in colon cancer cells is independent of the p53 status

**FIGURE 6:**
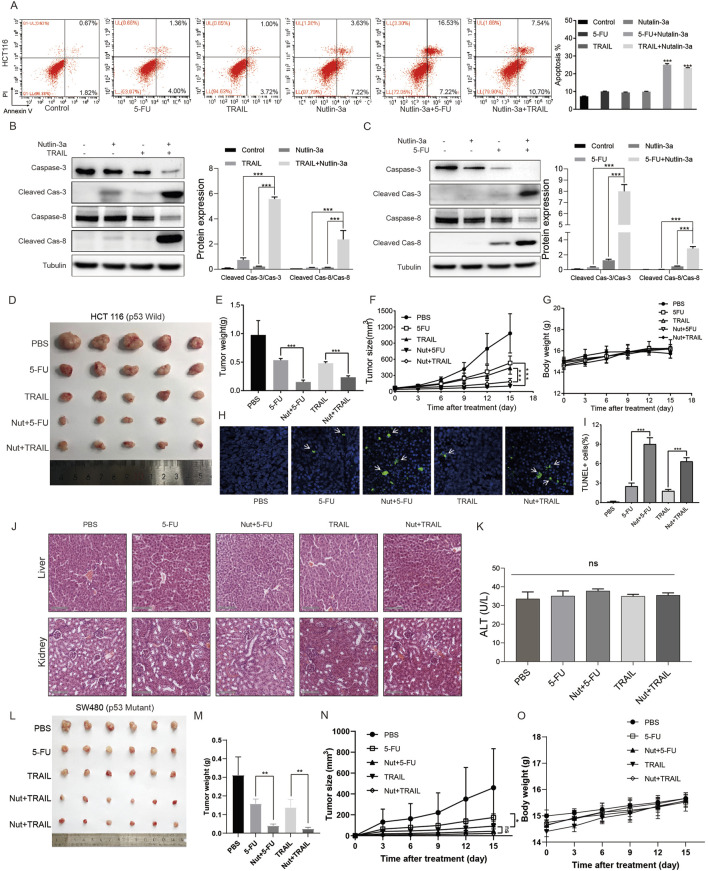
Nutlin-3a sensitized colon cancer cells to other anticancer agents *via* the death receptor pathway in *in vivo* tumor growth. **(A)** HCT116 cells were treated with TRAIL (15 μM), 5-FU (45 μM), nutlin-3a (50 μM), or their combination for 19 h, and the apoptosis rate was analyzed by flow cytometry. The percentages of apoptotic cells are presented in the histogram as the mean ± SD. **(B, C)** HCT116 cells were treated with TRAIL (15 μM), 5-FU (45 μM), nutlin-3a (50 μM), or their combination for 19 h, and cleaved caspase-3, cleaved caspase-8, and DR5 proteins were analyzed by Western blotting. Data are presented as the mean ± SD. **(D)** Nude mice were subcutaneously injected with 5 × 10^6^ HCT116 cells. After 7 days, the mice were treated with PBS, intraperitoneal injection of 5-FU (23 mg/kg/day), intratumoral injection of TRAIL (1 µg/2 days), or their combination with nutlin-3a (150 mg/kg twice daily) for 15 days. Xenograft tumors from each group were removed and photographed as shown. **(E)** Tumor weights were calculated and plotted (lower), n = 5 mice/group. **(F, G)** Tumor size and body weight of nude mice were monitored every 3 days during the 15 days of treatment. **(H, I)** Tumor tissues in different subgroups were analyzed by TUNEL staining. The ratio of TUNEL-positive cells is presented in the histogram as the mean ± SD. **(J)** HE staining and function analysis of the liver of the xenograft tumor model. **(K)** ALT, alanine aminotransferase; scale bars, 100 μm. Data are presented as mean + SD. *p < 0.05, **p < 0.01; ***, *p* < 0.01; n.s., not significant, by 1-factor ANOVA with a *post hoc* t-test **(D–F and K)**. **(L)** Nude mice were subcutaneously injected with 5 × 10^6^ SW480 cells. After 7 days, the mice were treated with PBS, intraperitoneal injection of 5-FU (23 mg/kg/day), intratumoral injection of TRAIL (1 µg/2 days), or their combination with nutlin-3a (150 mg/kg twice daily) for 15 days. Xenograft tumors from each group were removed and photographed as shown. **(M)** Tumor weights were calculated and plotted (lower), N = 5 mice/group. **(N, O)** Tumor size and body weight of nude mice were monitored every 3 days during the 15 days of treatment.

These results suggest that nutlin-3a can activate the death receptor signaling pathway by upregulating DR5. We hypothesized that nutlin-3a may sensitize colon cancer cells to TRAIL (DR5 ligand) or 5-FU (chemotherapeutic agents that induce DR5 via p53). First, the *in vitro* results showed that nutlin-3a can further increase TRAIL- and 5-FU-induced cell apoptosis through the death receptor apoptotic pathway (Figures 6A–C). In nude mice bearing colon cancer xenografts (wild-type p53: HCT-116, CT26; mutant p53: SW480), nutlin-3a further enhanced TRAIL- and 5-FU-induced tumor growth inhibition and apoptosis. Furthermore, we found that the combined treatment did not lead to significant changes in the body weight of the mice (HCT116: Figures 6D–I; CT26: Supplementary Figure S2A–D; SW480: Figures 6L–O). The examination of HE-stained tissue sections (Figure 6J) revealed that the combination therapy did not induce any morphological alterations or damage to the liver and kidneys. The histological analysis displayed an intact cellular structure, with hepatocytes showing normal architecture and no signs of necrosis, inflammation, or fibrosis in the liver. Similarly, renal tissue exhibited healthy glomeruli and tubules, with no evidence of glomerular damage or tubular degeneration. Furthermore, the assessment of hepatocyte-like transaminase (HLT) levels (Figure 6K) indicated that they remained within the normal physiological range, further corroborating the absence of liver or kidney dysfunction. These findings suggest that the combined administration of the drugs was well tolerated, with no significant adverse effects on the hepatic or renal pathology. These findings suggest that the combination of 5-FU and TRAIL with nutlin-3a may be a promising strategy for colon cancer therapies.

## 4 Discussion

As an E3 ubiquitin ligase, MDM2 can ubiquitinate and degrade a variety of tumor suppressor genes, including p53, Rb, p73, and ARF, which mediate the occurrence and development of a variety of tumors, including colorectal cancer (Chaudhary et al., 2020; Lau et al., 2008; Uchida et al., 2005; Hernandez-Monge et al., 2021). Small molecule inhibitors targeting MDM2 have become a new direction of targeted therapy, among which nutlin-3a is the most widely studied. Studies have shown that nutlin-3a can induce apoptosis of a variety of tumor cells; however, in a relatively low concentration of nutlin-3a (10–20 μM), colorectal cancer cells do not undergo apoptosis, showing obvious drug tolerance (Meijer et al., 2013; Zhou et al., 2018). In this study, we found that when the concentration of nutlin-3a exceeded 25 μM, a variety of colon cancer cells were induced to apoptosis.

It is worth noting that we found that nutlin-3a did not induce cell apoptosis through the classical endogenous mitochondrial apoptosis signaling pathway. The protein detection results showed that the expression level of antiapoptotic protein Bcl-2, which plays an important role in the mitochondrial apoptosis signaling pathway, was high in colon cancer cells. The administration of nutlin-3a did not inhibit Bcl-2 protein expression. On the other hand, activation of the mitochondrial pathway may require high concentrations to induce mitochondrial apoptosis in a short period of time, which may be the possible reason why nutlin-3a cannot activate the mitochondrial apoptosis pathway in a short period of time. Some reports indicate that antitumor drugs can induce cell apoptosis in the early stages by activating exogenous (death receptor) apoptosis signaling pathways. Ixazomib (Ninlaro), a proteasome inhibitor, can induce apoptosis in colorectal cancer cells by activating the DR5-mediated death receptor signaling pathway (Yue and Sun, 2019). Pseudolaric acid B induces apoptosis in head and neck cancer cells by activating caspase-8 (Choi et al., 2019). This raises the question of whether nutlin-3a can induce cell apoptosis through exogenous apoptotic signaling pathways. On the basis of these reports, we also detected the expression of caspase-8, an effector molecule of the death receptor apoptosis signaling pathway. Interestingly, we found that nutlin-3a can effectively activate caspase-8 activity in cells. Through different experiments and cell line screening, it was found that nutlin-3a mainly induces the activation of the death receptor pathway and apoptosis by upregulating DR5.

Based on the results of colon cancer cells with different p53 phenotypes, we found that nutlin-3a induces the expression of DR5 *via* a mechanism that appears to be independent of the p53 phenotype. This raises important questions regarding the interplay between p53-dependent and p53-independent pathways in the regulation of apoptosis in colorectal cancer cells. Studies have shown that there are two main types of DR5 activation in cells: one is the activation of p53 in response to DNA damage and the other is the activation of the ER stress condition CHOP gene (Dilshara et al., 2019; Taketani et al., 2012; Cao Y. et al., 2020; Byun et al., 2018; Chen et al., 2016).

p53 is well known for its role in cellular responses to stress, including DNA damage, where it promotes apoptosis through transcriptional upregulation of proapoptotic factors, such as DR5. However, our findings indicate that the elevation of DR5 in response to nutlin-3a occurs in both wild-type and mutant p53 backgrounds, suggesting that alternative signaling pathways may be at play. This phenomenon illustrates the concept of redundancy in cellular signaling, where multiple pathways can converge on similar biological outcomes.

The activation of the CHOP–DR5–caspase-8 pathway, independent of p53, further exemplifies this complexity. CHOP, as an ER stress-induced transcription factor, can activate DR5 expression, thereby facilitating extrinsic apoptotic signaling in response to ER stress. Studies have shown that multiple factors inside and outside the cell can lead to ER stress, and especially in recent years, there have been many reports on ER stress induced by antitumor agents (Bae et al., 2020; Xie et al., 2020; De La Chapa et al., 2019; Glab et al., 2017). When ER stress occurs, a series of signaling pathways will be activated through the unfolded protein response (UPR) to alleviate the occurrence of stress (Rozpedek et al., 2016). In this study, we show that nutlin-3a can significantly activate the ER stress-signaling pathway, thus further inducing the expression of CHOP. Interestingly, knocking down the expression of CHOP significantly reduced nutlin-3a-induced upregulation of DR5 and caspase-8. This finding aligns with that of previous studies, indicating that ER stress can promote cell death through mechanisms that do not necessarily involve p53. For instance, the induction of DR5 by rapalogs is mediated by the ER stress regulator and transcription factor CHOP but not the tumor suppressor p53, on rapid and sustained inhibition of 4E-BP1 phosphorylation, and it is attenuated by eIF4E expression (He et al., 2016).

Beyond CHOP, there are other key mediators and pathways involved in the UPR and ER stress response that could play crucial roles in nutlin-3a′s effects. For instance, the PERK (protein kinase RNA-like endoplasmic reticulum kinase) and IRE1 (inositol-requiring enzyme 1) pathways are important branches of the UPR (Ludwig et al., 2023). The activation of PERK leads to eIF2α phosphorylation, which reduces overall protein synthesis while selectively upregulating stress-responsive genes, including ATF4. The activation of the unfolded protein response using tunicamycin and thapsigargin was sufficient to activate the ATF4/CHOP stress-response axis and sensitize to nutlin-3. Furthermore, IRE1 activation contributes to XBP1 splicing, impacting the cell’s ability to manage protein folding and responses to stress (Yoshida et al., 2001). Investigating these pathways would provide a more comprehensive understanding of how nutlin-3a modulates ER function and contributes to its apoptotic effects.

Interestingly, reports have shown that the combined action of nutlin-3a and bortezomib can increase the concentration of calcium ions in the cytoplasm, leading to cell death (Lee et al., 2017). In this study, we found through experiments that different concentrations of nutlin-3a can individually cause an increase in cytoplasmic calcium ion concentration, which occurs earlier than the time of cell death. This suggests that nutlin-3a mediates CHOP-dependent cell death by affecting the concentration of calcium ions in the ER, leading to a transitional ER stress response.

Numerous reports have shown that there is a calcium ion correlation between the ER and mitochondria (Marchi et al., 2018; Janer et al., 2023; Wu et al., 2023). Calcium ions released from the endoplasmic reticulum can be taken up by mitochondria, and the transition of calcium ions taken up by mitochondria can lead to mitochondrial-dependent cell death and apoptosis. Although nutlin-3a we previously detected did not affect mitochondrial apoptosis proteins and pathways, this does not rule out the possibility that mitochondrial-dependent apoptosis may occur in the later stages of endoplasmic reticulum stress apoptosis and death receptor apoptosis pathways with prolonged action time.

The major challenge in the clinical use of nutlin-3a is drug resistance, including both primary and acquired resistance. Understanding the mechanism of drug tolerance and finding effective drug combinations are crucial for providing drug responsiveness in patients. The death receptor signaling pathway mediated by ER stress induced by nutlin-3a provides a direction for drug combination. The coexistence of p53-dependent and p53-independent mechanisms highlights the potential for therapeutic strategies that can target these pathways synergistically. Combining MDM2 inhibitors, such as nutlin-3a, with agents that further activate the death receptor pathway or ER stress may enhance overall efficacy, particularly in those exhibiting p53 pathways’ dysfunction. As we know, TRAIL can bind to DR4 and DR5, which further activate FADD (Fas-associated protein with death domain) and caspase-8 (Thapa et al., 2020; Yuan et al., 2018). 5-FU, as a commonly used chemotherapy drug for colorectal cancer, has been reported to upregulate the expression of DR5 in cells through p53, inducing the activation of exogenous apoptosis signaling pathways (Longley et al., 2006; Yu et al., 2013). Based on the mechanism of action of TRAIL and 5-FU, we chose to use nutlin-3a in combination with TRAIL and 5-FU, which showed a significant inhibitory effect on growth *in vitro* and *in vivo*. Importantly, our expanded *in vivo* studies using three independent colon cancer models (HCT116 and CT26 with wild-type p53; SW480 with mutant p53) conclusively show that nutlin-3a-mediated chemosensitization is not restricted by the p53 status. This finding highlights its potential to overcome resistance in tumors with dysfunctional p53 pathways, which is a common feature of advanced colorectal cancers.

In addition, Ludwig et al. found that the activation of the unfolded protein response, whether using tunicamycin or thapsigargin, can enhance the sensitivity of liposarcoma cells to nutlin-3a (Ludwig et al., 2023). Marco Cordani et al. suggest that thapsigargin may facilitate the degradation of mutant Tp53 (MUT Tp53) when macroautophagy is activated (Yoshida et al., 2001). Based on these considerations, tunicamycin may be a more effective ER stress inducer for maximizing the degradation of MUT Tp53 and minimizing its oncogenic effects. The combination of nutlin-3a and tunicamycin may have good prospects in the treatment of colorectal cancer.

Although the study mentions some new mechanisms of nutlin-3a in colorectal cancer cells, there are still many issues that need further experimental solutions and clarification. Colorectal cancer cells with different p53 phenotypes can cause similar phenomena, but there are still certain differences in death receptor-related apoptosis induced by different drug concentrations. The research on the specific signaling pathway of nutlin-3a regulation of endoplasmic reticulum stress is not deep enough, and it is necessary to use different p53 subtypes of colorectal cancer cells to further clarify the universality of nutlin-3a regulation of ER stress. Only CCK8 was used to detect cell death. Other detection methods, such as LDH detection, require further clarification of the relevant concentration of action. In terms of combination therapy, can ER stress activators really further increase nutlin-3a-induced cell death? In addition, there should be further studies regarding whether the increase in cytoplasmic calcium ion concentration caused by nutlin-3a will further affect mitochondrial function or cell death caused by mitochondrial calcium overload.

## 5 Conclusion

**FIGURE 7:**
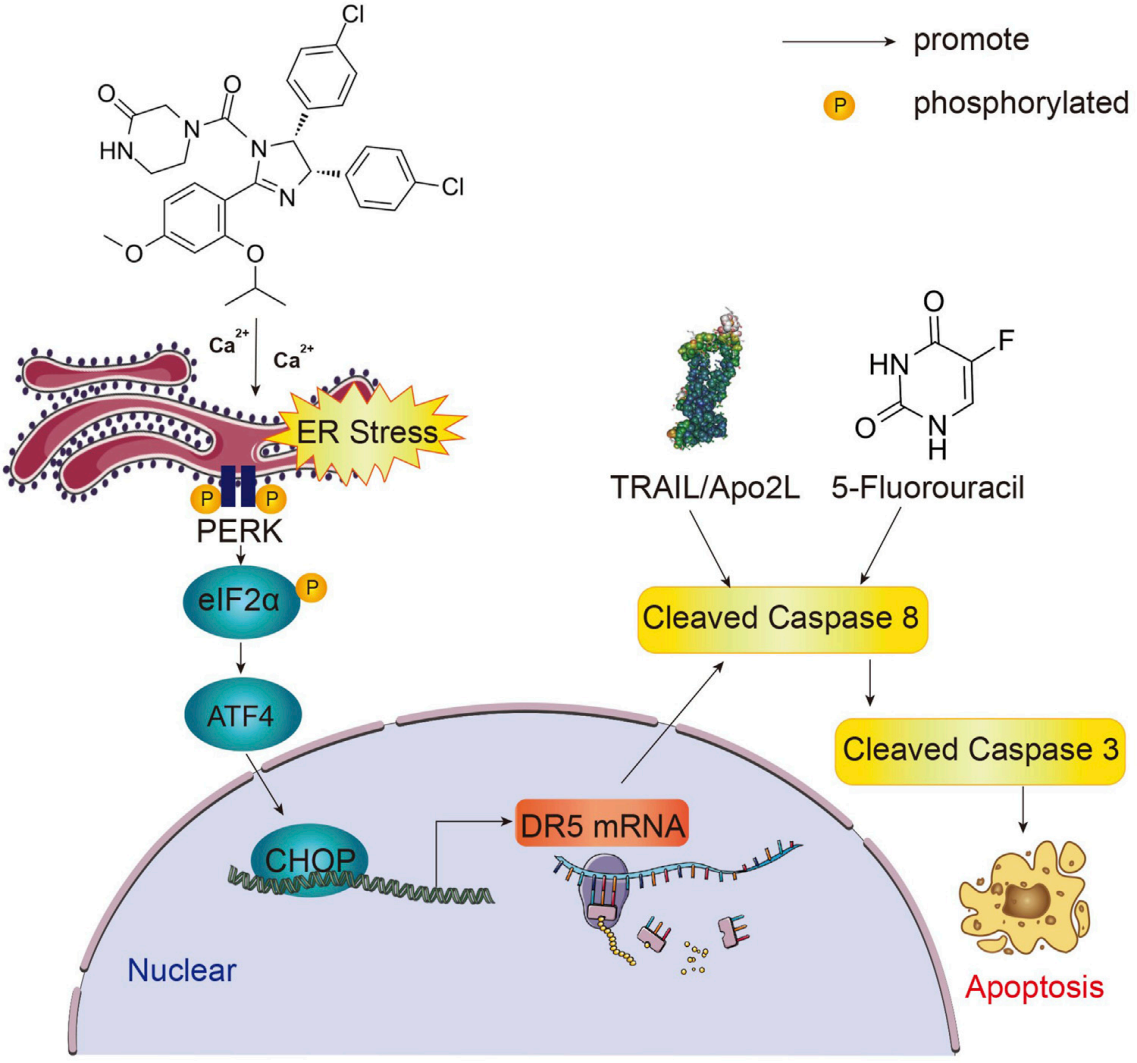
Graphical summary of the study. Nutlin-3a induces ER stress and CHOP upregulation by increasing the cytosolic calcium concentration. In the nucleus, by regulating the transcription of DR5, CHOP can activate the initiating caspase family protein caspase-8. By activating the initiating caspase family, nutlin-3a enhances drug sensitivity to 5-FU and TRAIL and induces apoptosis in p53 wild-type and mutant colon cancer cells.

In this present study, we demonstrated that nutlin-3a inhibits colon cancer *in vivo* and *in vitro* by triggering CHOP/DR5/caspase-8-dependent apoptosis. Nutlin-3a induces stress by promoting the release of Ca^2+^ from the ER, thereby upregulating CHOP. Nutlin-3a synergistically enhanced the efficacy of clinical chemotherapeutic agents TRAIL and 5-FU in both wild-type and mutant p53 colon cancer models (Figure 7).

## Data Availability

The original contributions presented in the study are included in the article/supplementary material; further inquiries can be directed to the corresponding authors.
