# Supplementary material for: MDM2 inhibitor induces apoptosis in colon cancer cells through activation of the CHOP-DR5 pathway, independent of p53 phenotype
Source: Front Pharmacol. 2025 Apr 8;16:1508421. doi: 10.3389/fphar.2025.1508421 (PMC12011796; doi:10.3389/fphar.2025.1508421)
Supplement: Supplementary file 1 [file Table1.docx]

Table S1 The list of primer sequences that used for RT-PCR analysis

| Gene | Primer | Sequence |
| --- | --- | --- |
| GAPDH | Forward | 5'-AGAAGGCTGGGGCTCATTTG-3' |
|  | Reverse | 5'-AGGGGCCATCCACAGTCTTC-3' |
| DCR1 | Forward | 5'-CCTTCTTGCTTCCCATGTAC-3' |
|  | Reverse | 5'-CTTCAACACACTGGATATCATC-3' |
| DCR2 | Forward | 5'-TCATGTCCTTCACGAGTTCC-3' |
|  | Reverse | 5'-CTCTGACACCCTTCAGCTTC-3' |
| DCR3 | Forward | 5'-CCTCAATGTGCCAGGCTCTT-3' |
|  | Reverse | 5'-ATGACGGCACGCTCACACT-3' |
| DR4 | Forward | 5'-CTCGCTGTCCACTTTCGTCTC-3' |
|  | Reverse | 5'-GTACCAGCTCTGACCACATC-3' |
| DR5 | Forward | 5'-AAGACCCTTGTGCTCGTTGT-3’ |
|  | Reverse | 5'-AGGTGGACACAATCCCTCTG-3’ |
| TRAIL | Forward | 5'-GAGCTGAAGCAGATGCAGGAC-3' |
|  | Reverse | 5'-TGACGGAGTTGCCACTTGACT-3' |
| FAS | Forward | 5'-TGTAGATTGTGTGATGAAGG-3' |
|  | Reverse | 5'-GATTCCATGTTCACATTTGG-3' |
| TNFR1 | Forward | 5'-TGTGCACCTGCCATGCAGG-3' |
|  | Reverse | 5'-CAGCACTGTGGTGCCTGAG-3' |
| TNFR2 | Forward | 5'-GCCCCACCAGATCTGTAACGTG-3' |
|  | Reverse | 5'-TGAGGCACCTTGGCTTCTCTC-3' |
| CHOP | Forward | 5'-GAACCAGGAAACGGAAACAG-3 |
|  | Reverse | 5'-ACCATTCGGTCAATCAGAGC-3 |
| BIP | Forward | 5'-ATCATCAACGAGCCTACGG-3' |
|  | Reverse | 5'-ACACGCTGGTCAAAGTCTTCT-3' |
| GADD34 | Forward | 5'-ACCTCTACTTCTGCCTTGTCTCC-3' |
|  | Reverse | 5'-TGGCTCCTTTACTTCTTTCTGTT-3' |
