# Supplementary figures and images for: MDM2 inhibitor induces apoptosis in colon cancer cells through activation of the CHOP-DR5 pathway, independent of p53 phenotype
Source: Front Pharmacol. 2025 Apr 8;16:1508421. doi: 10.3389/fphar.2025.1508421 (PMC12011796; doi:10.3389/fphar.2025.1508421)

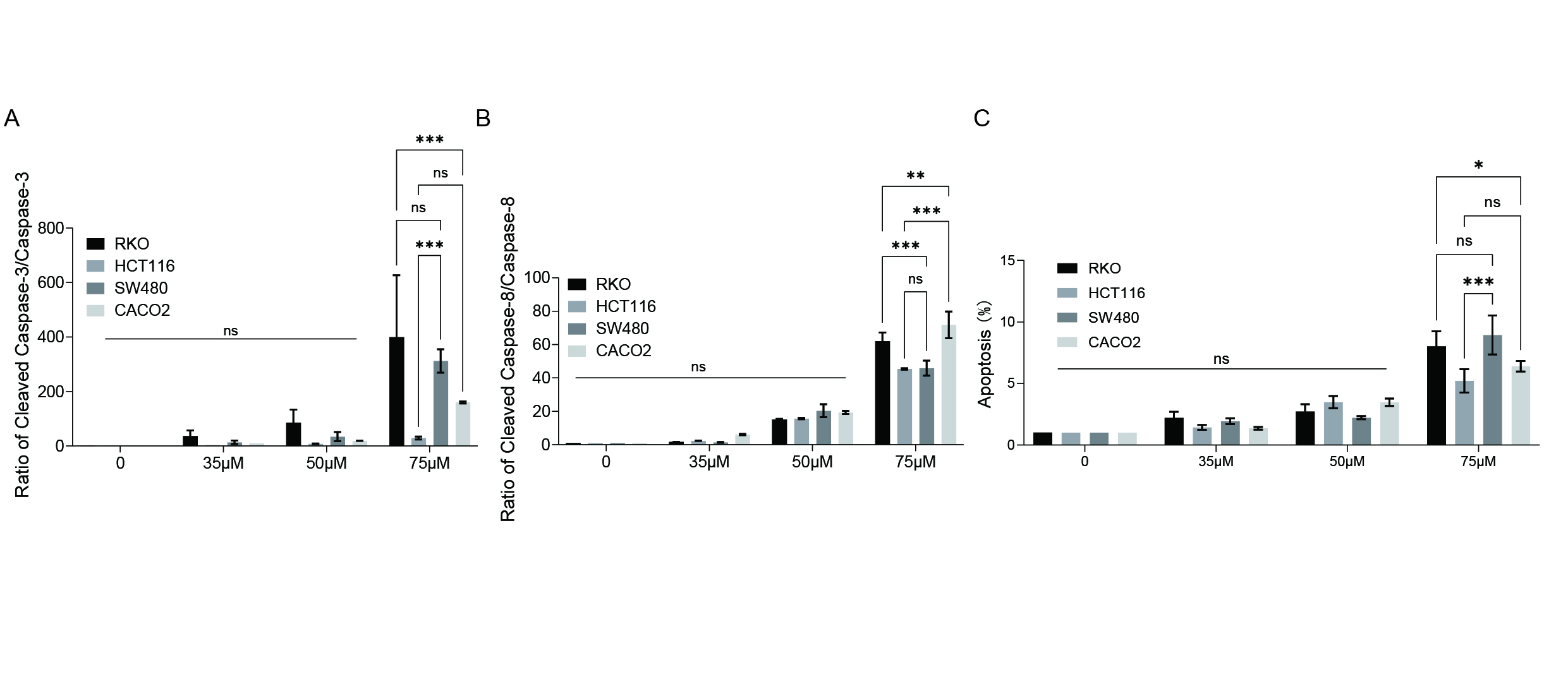

Supplement: Supplementary file 2 [file Image1.jpeg]

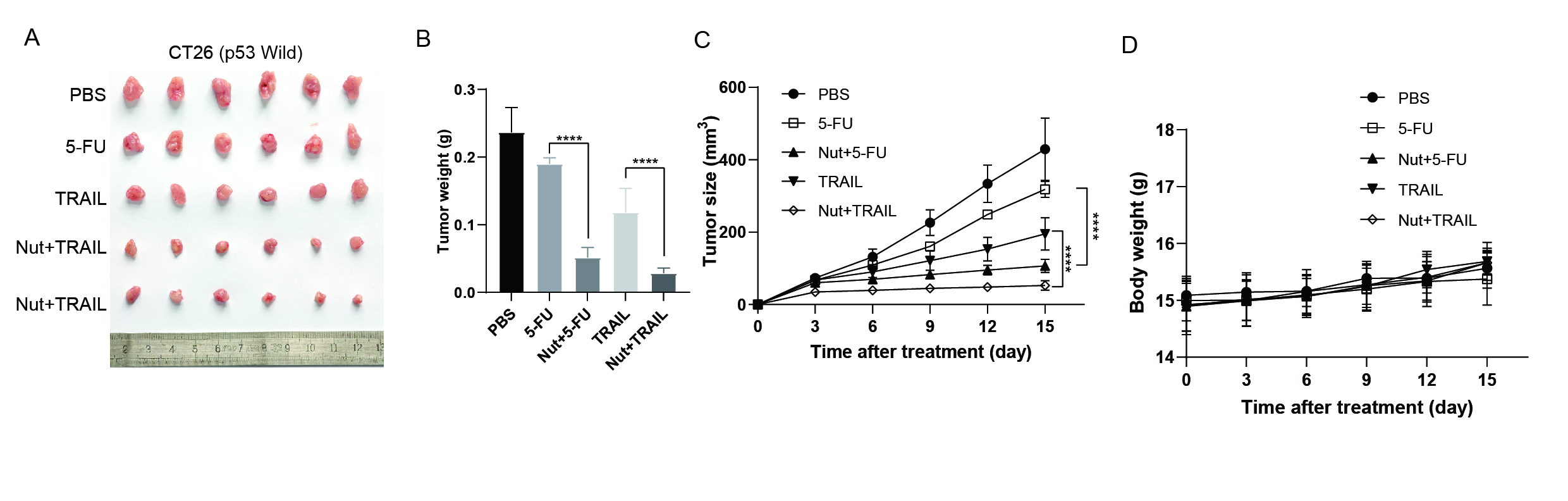

Supplement: Supplementary file 3 [file Image2.jpeg]
